# Robust estimates of heritable coronary disease risk in individuals with type 2 diabetes

**DOI:** 10.1002/gepi.22434

**Published:** 2021-10-21

**Authors:** Christopher Grace, Jemma C. Hopewell, Hugh Watkins, Martin Farrall, Anuj Goel

**Affiliations:** ^1^ Division of Cardiovascular Medicine, Radcliffe Department of Medicine University of Oxford Oxford UK; ^2^ Wellcome Centre for Human Genetics University of Oxford Oxford UK; ^3^ Nuffield Department of Population Health University of Oxford Oxford UK

**Keywords:** genetics, Mendelian randomization, metabolic syndrome

## Abstract

Type 2 diabetes (T2D) is an important heritable risk factor for coronary artery disease (CAD), the risk of both diseases being increased by metabolic syndrome (MS). With the availability of large‐scale genome‐wide association data, we aimed to elucidate the genetic burden of CAD risk in T2D predisposed individuals within the context of MS and their shared genetic architecture. Mendelian randomization (MR) analyses supported a causal relationship between T2D and CAD [odds ratio (OR) = 1.13 per log‐odds unit 95% confidence interval (CI): 1.10–1.16; *p* = 1.59 × 10^−17^]. Simultaneously adjusting MR analyses for the effects of the T2D instrument including blood pressure, dyslipidaemia, and obesity attenuated the association between T2D and CAD (OR = 1.07, 95% CI: 1.04–1.11). Bayesian locus‐overlap analysis identified 44 regions with the same causal variant underlying T2D and CAD genetic signals (FDR < 1%) at a posterior probability >0.7; five (MHC, *LPL, ABO, RAI1* and *MC4R*) of these regions contain genome‐wide significant (*p* < 5 × 10^−8^) associations for both traits. Given the small effect sizes observed in genome‐wide association studies for complex diseases, even with 44 potential target regions, this has implications for the likely magnitude of CAD risk reduction that might be achievable by pure T2D therapies.

## 1 INTRODUCTION

The pandemic (Unnikrishnan et al., 2017; Zheng et al., 2018) of type 2 diabetes (T2D) is having an increasing impact on morbidity and mortality in high and medium income countries, with over 1 million deaths per year (Khan et al., 2020). The diagnostic high blood glucose levels that result from lack of insulin production by the pancreatic islets, or insulin resistance, put patients at risk of micro‐vascular diseases such as nephropathy and retinopathy. Diabetics are also at increased risk of macro‐vascular diseases including coronary artery disease (CAD), one of the leading causes of mortality in the world with 8.9 million deaths in 2017 (Cade, 2008; “Global, regional, and national age‐sex‐specific mortality for 282 causes of death in 195 countries and territories, 1980‐2017: a systematic analysis for the Global Burden of Disease Study 2017”, 2018; Sarwar et al., 2010). An extensive literature review (Einarson et al., 2018) reported that one‐fifth of patients with T2D also suffer from CAD; a similar trend was observed in the United Kingdom based on primary care data (Lautsch et al., 2019). This comorbid connection is supported by recent drug trials of compounds that lower blood glucose levels, which report a reduction in the number of CAD‐associated events as a welcome side benefit of these diabetes therapies. For instance, the alpha‐glucosidase inhibitor acarbose (Chiasson et al., 2003) is associated with a substantial (49%) relative risk reduction [hazard ratio: 0.51, 95% confidence interval (CI): 0.28–0.95, *p* = 0.03] of cardiovascular events in patients with T2D (including peripheral vascular disease, stroke, heart failure) compared with placebo. Empagliflozin, an inhibitor of sodium–glucose cotransporter 2 (Zinman et al., 2015), is reported to have a lower rate (14% risk reduction) of primary cardiovascular events, a composite of death from cardiovascular causes as well as nonfatal myocardial infarction or nonfatal stroke, compared with standard treatment. Although these risk reductions are clinically welcome, they may follow concomitant reductions in other CAD risk factors, for example, blood pressure, rather than reflecting some direct normoglycaemic mechanism.

T2D and CAD are typical common complex diseases with substantial polygenic components that have been examined in numerous genome‐wide association studies (GWAS); the most recent published large‐scale GWAS meta‐analysis reported 243 and 161 genome‐wide significant (GWS) independently associated variants associated with T2D (Mahajan et al., 2018) and with CAD respectively (van der Harst & Verweij, 2018). Such data provide opportunities to probe for shared heritable, and thus potentially causal, links between diseases. For instance, based on earlier data, Bulik‐Sullivan et al. reported a genetic correlation of 0.39 (95% CI: 0.24–0.53) between T2D and CAD in a linkage disequilibrium (LD) score regression analysis (Bulik‐Sullivan et al., 2015). Contemporaneous attempts (Ahmad et al., 2015; Benn et al., 2012; Gan et al., 2019; Jansen et al., 2015; Merino et al., 2017; Ross et al., 2015; Tikkanen et al., 2016; Zhao et al., 2017) to compare genetically and epidemiologically measured risk found variable increases in CAD risk (up to 1.63‐fold per T2D log‐odds) reflecting shared inherited and potentially environmental susceptibility. However, understanding the intricate relationship of obesity, lipid traits and hypertension with T2D and CAD remains a challenge that has only been partially resolved (Goodarzi & Rotter, 2020).

It is becoming increasingly apparent that variants associated with common complex diseases like T2D and CAD can show pleiotropic associations with other heritable phenotypes, specifically metabolic syndrome (MS) (Eckel et al., 2005; Kaur, 2014) traits such as obesity or dyslipidaemia (Hackinger & Zeggini, 2017), revitalizing the ‘common soil’ hypothesis (Stern, 1995). In this study, we have probed the extensive new GWAS meta‐analysis data (Mahajan et al., 2018; van der Harst & Verweij, 2018), to explore the nature of the link between T2D and CAD heritable risk. We have applied a series of complementary statistical genetic methods based upon genetic association summary statistics, including Mendelian randomization (MR) analysis taking into account pleiotropic metabolic confounders. We also performed a systematic locus‐by‐locus assessment of T2D and CAD to quantify and identify the loci that are driven by the same likely causal variant. These common driver loci can give clues to intersecting pathways between the two diseases.

## 2 METHODS

### 2.1 Participating cohorts

T2D genetic association summary statistics (Mahajan et al., 2018) were extracted from a meta‐analysis (unadjusted for BMI) of 32 GWAS studies comprising 74,124 cases and 824,006 controls of European ancestry, performed by the DIAGRAM consortium. Briefly, T2D case status was established by means of patient questionnaire and medical record data, with an age‐of‐onset >35 years and exclusion of glutamic acid decarboxylase (GAD) antibody‐positive individuals.

CAD genetic association summary statistics (van der Harst & Verweij, 2018) were extracted from a meta‐analysis of UK Biobank and CARDIoGRAMplusC4D (Nikpay et al., 2015) 1000 Genomes GWAS (involving 48 studies) that in total included 122,733 cases and 424,528 controls. Case status included various CAD diagnoses; myocardial infarction, acute coronary syndrome, chronic stable angina with a revascularization procedure and angiographic evidence of >50% coronary stenosis. CAD cases had a mean age of approximately 60 years.

Genetic association summary statistics for MS traits [body mass index (BMI) (Yengo et al., 2018), waist‐hip ratio (WHR) (Pulit et al., 2019), systolic and diastolic blood pressure (SBP, DBP) (Evangelou et al., 2018), fasting blood glucose (without diabetics) (Dupuis et al., 2010) and serum lipids (Teslovich et al., 2010) (high‐density lipoprotein (HDL), triglycerides (TG)] were downloaded from public websites (Table S1).

This study constitutes a *de novo* analysis of anonymized summary‐level genetic data from published meta‐analyses in which each of the contributing studies secured ethics approval from their respective authorities; all participants had provided written informed consent (Mahajan et al., 2018; Nikpay et al., 2015; van der Harst & Verweij, 2018).

### 2.2 Cross‐trait LD score regression

Alleles and direction of susceptibility effect (scaled as log odds‐ratios) for the T2D and CAD data sets were aligned to the Hapmap3 set of 1.2 million variants. The genetic correlation of variant effects between T2D and CAD was then estimated using cross‐trait LD score regression (Bulik‐Sullivan et al., 2015) using European LD scores computed using 1000 Genomes data bundled with the software.

### 2.3 Selection of instrumental variables

243 lead variants with genome‐wide significant associations (i.e., *p* < 5 × 10^−8^) were extracted from the DIAGRAM meta‐analysis, log odds‐ratio estimates (betas) and effect/reference allele identities were collated for our analysis. Among the 243 variants identified, 224 were selected as instrumental variables (IVs) (Mahajan et al., 2018) for this study; proxies (LD *r* ^2^ > 0.8) for the remaining 19 T2D variants were unavailable in the CAD meta‐analysis (Table S2). Per single‐nucleotide polymorphism (SNP) estimates of liability heritability for IVs were calculated using the INDI‐V online tool (Witte et al., 2014) assuming a 10% population prevalence for T2D. *F*‐statistics to assess the strength of IVs were calculated using the formula $F=\left(\frac{n-k-1}{k}\right)\left(\frac{R^{2}}{1-\;R^{2}}\right)$ where *n* = sample size, *k* = the number of IVs, and *R* ^2^ = the total SNP heritability summed across K IVs (Burgess & Thompson, 2011; Cragg & Donald, 1993).

Summary statistics (betas and their standard errors and effect/reference allele identity) of the IVs for MS CAD risk factors (BMI, WHR, SBP, DBP, fasting blood glucose and serum lipids (HDL and TG)) were aligned (i.e., ‘beta flipped’) to the increasing T2D risk allele for MR analyses (Tables S2 and S5).

### 2.4 Instrumental variable sensitivity analysis

The PhenoScanner database (Kamat et al., 2019; Staley et al., 2016) was interrogated for evidence of pleiotropy between T2D associated variants and MS (Eckel et al., 2005) traits; obesity, blood pressure, glucose and lipids, with a p‐value threshold of 1 × 10^−5^ and a LD *r* ^2^ threshold of 0.8 to identify proxy variants (Tables S3 and S4). In total, 93 of 224 IVs showed evidence of pleiotropy with at least one metabolic trait and were removed in the sensitivity analysis.

### 2.5 MR analysis

The causal effect of T2D (in the role of risk phenotype) on CAD (as outcome phenotype) was first analysed in a standard, random‐effect (Jack Bowden et al., 2017), inverse variance weighted (IVW) (Burgess et al., 2013) MR. This univariate method performs a meta‐analysis of the ratios of the outcome (i.e., CAD) variant effect sizes divided by the risk phenotype (i.e., T2D) effect sizes; standard errors (SEs) of the outcome effect sizes were used as weights. These ratios provide causal estimates for the effect of the risk factor on the outcome.

IVW assumes that all IVs used in the analysis do not violate IV assumptions. To test the validity of the IVs, sensitivity analyses were performed using the MR Egger (Bowden et al., 2015), weighted median (Bowden et al., 2016), weighted mode (Hartwig et al., 2017) and MRPRESSO (Verbanck et al., 2018) methods. MR Egger (Bowden et al., 2015; Burgess & Thompson, 2017) detects invalid IVs due to directional pleiotropy, (where the IV influences both the risk and outcome phenotypes directly) or violation of the InSIDE assumption (or both). The InSIDE assumption is that the direct effect of the IV on the exposure is independent on the direct effect on the outcome and must be true for the MR Egger test to be valid (Bowden et al., 2015). The weighted median (Bowden et al., 2016) provides a robust estimator of causal effect that allows for up to 50% of the IVs being invalid; the median MR method is insensitive to outlier outcome betas. The weighted mode (Hartwig et al., 2017) method has been shown to be robust if the majority of the IVs are valid (Hartwig et al., 2017). MR‐PRESSO (Verbanck et al., 2018) detects and corrects for outliers with horizontal pleiotropy within the list of Instrumental variables.

We extended the MR using 176 IVs/proxy IVs available in all of the MS traits to simultaneously evaluate single or multiple MS traits (Evangelou et al., 2018; Pulit et al., 2019; Teslovich et al., 2010; Yengo et al., 2018) with T2D in a CAD risk outcome model (Table S5). A stepwise model selection procedure using the Akaike information criterion (AIC) (Akaike, 1974) was performed to identify a parsimonious subset of informative risk factors to be included with T2D in the MR model. This method takes a saturated model (including all MS traits), and finds the model that minimizes the AIC estimator by systematically removing and adding traits from the model. AIC is derived from the likelihood score of the model.

To perform the MR analysis we used the R package TwoSampleMR version 0.5.5 with R version 4.0.3. The stepwise model selection used the R stats package version 4.03.

### 2.6 Shared genetic determinants between T2D and CAD

The LDetect (Berisa & Pickrell, 2016) method was used to split the genome into 1,703 discrete segments that are in approximate linkage equilibrium based on European participants in the 1000 Genomes database (Auton et al., 2015). The *gwas‐pw* (Pickrell et al., 2016) method was used to undertake a Bayesian analysis to assess whether T2D and CAD share a common underlying genetic signal. This method calculates the likelihood whether a genomic region contains a variant influencing only T2D susceptibility (Model 1), only CAD (Model 2), a shared variant that influences both T2D and CAD (Model 3) or contains two independently associated variants (Model 4). Loci reporting a posterior probability of association in Model 3 (PPA3) > 0.7 and harbouring suggestive (FDR < 1%) T2D and CAD associations of variant were assembled. The FDR < 1% threshold was calculated from the T2D summary GWAS using Simes method (Stata10.1), the corresponding *p*‐value threshold was *p* < 2.2 × 10^−5^; this threshold also controls the CAD GWAS FDR to <1%.

## 3 RESULTS

### 3.1 Genome‐wide genetic correlation between T2D and CAD

A cross‐trait LD score regression analysis based on a Hapmap 3 set of genome‐wide variants detected a highly significant (*p* < 3.11 × 10^−75^) genetic correlation between CAD and T2D of moderate strength (*r*_g_ = 0.40, 95% CI: 0.36–0.44).

### 3.2 MR analysis—causal role of T2D on CAD

Analysis of 224 IVs in a standard random‐effect IVW MR test showed a highly significant (*p* < 1.59 × 10^−17^) association with an odds ratio (OR) of 1.13 for CAD per log odds unit of T2D susceptibility (Table 1, Figure S1). The T2D GWAS was based on an effective sample size of 231,456 participants (Mahajan et al., 2018), the total SNP heritability for the 224 instruments and the corresponding *F*‐statistic were calculated as 12.4% and 146.1, respectively. An Egger horizontal pleiotropy test was significant (*p* < 0.003) suggesting a violation of the IV assumption due to directional pleiotropy (that the genetic variant does not directly influence the outcome phenotype) (Figure S2). The ORs estimated by the weighted median and mode MR methods, tests that are partially robust to pleiotropy, were very similar (OR = 1.09), but slightly attenuated in comparison with the IVW effect size (Table 1).

**Table 1:** Results of the T2D‐CAD MR analysis and senstitivity tests

	MR test	OR for CAD [95% CI]	*p*‐value	IVs (*n*)
All IVs	IVW—fixed	1.13 [1.11−1.14]	2.78 × 10^−71^	224
	IVW—random	1.13 [1.10−1.16]	1.59 × 10^−17^	
	Weighted median	1.09 [1.06−1.12]	6.05 × 10^−9^	
	Weighted mode	1.09 [1.06−1.12]	1.28 × 10^−7^	
Pleiotropic IVs	IVW—fixed	1.10 [1.08−1.12]	6.48 × 10^−19^	131
Removed	IVW—random	1.10 [1.06−1.14]	1.86 × 10^−7^	
	Weighted median	1.07 [1.03−1.10]	2.09 × 10^−4^	
	Weighted mode	1.06 [0.99−1.14]	0.11	

*Note*: See Tables S3 and S4 for details of the pleiotropic phenotypes and variants. *n* denotes the number of IVs.

Abbreviations: CAD, coronary artery disease; CI, confidence interval; IV, instrumental variant; IVW, inverse variance weighted; MR, Mendelian randomization; OR, odds ratio; T2D, type 2 diabetes.

### 3.3 Sensitivity analyses—excluding pleiotropic variants

When pleiotropic variants identified by PhenoScanner were removed, the IVW signal was marginally weaker (OR = 1.10, 95% CI: 1.06–1.14) although still highly significant (*p* < 1.86 × 10^−7^) (Table 1, Figure S3); the total SNP heritability for the 131 pleiotropy‐filtered instruments was 5.6% with an *F*‐statistic = 104.7. MR Egger pleiotropy test was again significant (*p* < 0.03) indicating the presence of residual directional pleiotropy (Figure S4). OR estimates from the weighted median was marginally smaller (OR = 1.07, 95% CI: 1.03–1.10, *p* < 2.09 × 10^−4^).

MR Egger analyses suggest that some of the IVs used in this analysis violate the null directional pleiotropy IV assumption. The difference between the standard IVW and weighted median and mode MR odds‐ratios, indicate that some of the IVs are invalid due to either horizontal pleiotropy or mutual confounding.

MR‐PRESSO identified 12 variants that were significantly heterogeneous and after excluding the outlier instruments, the results were consistent with standard IVW (OR = 1.14, 95% CI: 1.11–1.16, *p* < 2.51 × 10^−24^) (Table S6).

### 3.4 MR analysis of T2D and associated metabolic traits

To investigate potential conflation of T2D and associated MS genetic influences on CAD risk, the IVW MR model was expanded to simultaneously test multiple traits. The effect of a common set of IVs (*n* = 176, SNP‐heritability = 8.5%, *F*‐statistic = 122.5) was estimated for T2D in conjunction with one or several additional traits using a weighted multiple regression model with the intercept fixed at zero. When individual MS traits were included in the model, T2D causal estimates fell in the range OR = 1.10–1.15 (Table 2), these estimates are similar to that derived from the standard (univariate) IVW MR analysis (Table 1). The influence of MS traits on CAD showed consistent directional effects, that is, HDL being protective, OR = 0.74, while the others showed deleterious effects. Significant ORs were observed for all of the MS traits except for DBP and glucose (*p* > 0.35).

**Table 2:** Bi‐variate MR analyses

	Trait	Trait		T2D effect on CAD		MS trait effect on CAD	
MS trait	*h* ^2^	*F*	MS units	OR [95% CI]	*p*‐value	OR [95% CI]	*p*‐value
DBP	0.21	9.1	mm Hg	1.13 [1.10–1.17]	3.68 × 10^−15^	1.01 [0.99–1.04]	0.353
SBP	0.21	9.1	mm Hg	1.11 [1.08‐1.15]	5.97 × 10^−11^	1.02 [1.01–1.04]	8.38 × 10^−3^
HDL	1.46	8.4	per SD	1.10 [1.07–1.13]	2.32 × 10^−10^	0.74 [0.66–0.84]	2.14 × 10^−6^
TG	2.02	11.7	per SD	1.11 [1.08–1.14]	4.15 × 10^−14^	1.41 [1.28–1.54]	6.17 × 10^−12^
BMI	1.27	58.2	per SD	1.13 [1.10–1.16]	8.13 × 10^−15^	1.16 [1.02–1.32]	0.023
WHR	0.63	25.0	per SD	1.11 [1.08–1.15]	1.14 × 10^−10^	1.38 [1.13–1.68]	1.93 × 10^−3^
Glucose	2.48	6.7	mmol/l	1.15 [1.11–1.19]	1.13 × 10^−12^	0.91 [0.75–1.12]	0.381

*Note*: Analysis was performed using 176 instrumental variables of individual MS traits in addition to T2D. *h* ^2^ reports the heritability and *F* reports the strength of the IVs used for the given MS trait. T2D effect on CAD denotes the IVW MR estimate of the causal effect of T2D on CAD, when a single MS trait is added to the model. MS trait effect on CAD: The estimate of the effect (scaled in MS units) of the MS trait on CAD risk.

Abbreviations: BMI, body mass index; CAD, coronary artery disease; CI, confidence interval; DBP, diastolic blood pressure; Glucose, fasting glucose; HDL, high‐density lipoprotein cholesterol; MR, Mendelian randomization; MS, metabolic syndrome; OR, odds ratio; SBP, systolic blood pressure; T2D, type 2 diabetes; TG, triglycerides; WHR, waist‐hip ratio.

In addition to testing the effect of including individual MS traits, we assessed the impact of multiple traits in a multiple regression model (Table 3). Two models were analysed; a saturated model (Table S7a) including all MS traits and a parsimonious model (Table 7b) identified by applying a stepwise model selection procedure and the Akaike information criterion (AIC) (Table S7c). The T2D causal estimates were very similar in both the saturated (OR = 1.07, *p* = 0.002) and parsimonious model (OR = 1.07, *p* = 9.7 × 10^−6^) and similar to those estimated in the weighted median and mode analyses (Table 1).

**Table 3:** Results of a parsimonious MR model selection procedure minimizing the Akaike information criterion (AIC)

Covariate	Units	OR [95% CI]	*p*‐value
T2D	per log odds	1.07 [1.04–1.11]	9.73 × 10^−6^
TG	per SD	1.32 [1.20–1.45]	2.67 × 10^−8^
BMI	per SD	1.18 [1.06–1.33]	4.82 × 10^−3^
WHR	per SD	1.25 [1.03–1.51]	2.65 × 10^−2^
SBP	mm Hg	1.03 [1.00–1.05]	3.05 × 10^−2^
DBP	mm Hg	0.97 [0.94–1.01]	1.43 × 10^−1^

Abbreviations: BMI, body mass index; CI, confidence interval; DBP: diastolic blood pressure; MR, Mendelian randomization; OR, odds ratio; SBP, systolic blood pressure; T2D, type 2 diabetes; TG, triglycerides; WHR, waist‐hip ratio.

The parsimonious model included representative traits from the three pillars of MS, blood pressure, dyslipidaemia and obesity, reflecting the correlation between closely related phenotypes (Tables 3 and S7b).

**Figure 1:**
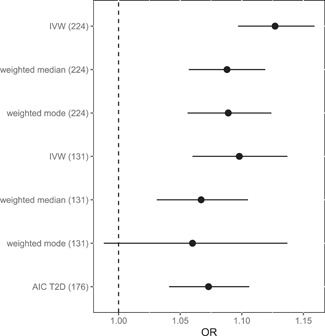
Comparison of causal effects of T2D on CAD. IVW (224) is the causal estimate derived from a univariate random‐effect IVW MR analysis including 224 IVs. IVW (131) is the univariate causal estimate for 131 IVs non‐pleiotropic variants. AIC T2D is the causal estimate from the parsimonious MR with MS covariates model after AIC stepwise selection. Weighted median and weighted mode are the corresponding sensitivity analyses for the 224 and 131 analyses. AIC, Akaike information criterion; CAD, coronary artery disease; IV, instrumental variable; IVW, inverse variance weighted; MR, Mendelian randomization; T2D, type 2 diabetes

Comparing the effect of T2D IVs on CAD using different models, we detected a significant causal association ranging from OR = 1.13 using all IVs to OR = 1.07 after model selection (Figure 1).

### 3.5 Pair‐wise comparison of loci—GWAS‐PW analysis

Of the 1,703 approximately genetically independent regions showing association, there were 47 regions harbouring both T2D and CAD GWS variant(s) (Table S8). A simulation with 100,000 permutations estimated the unbiased *p*‐value for ≥ 47 overlaps as *p* < 3 × 10^−5^ (Figure S5) suggesting there are more overlapping GWS loci than by chance. A *gwas‐pw* analysis suggested that only 5 out of 47 GWS overlapping loci (MHC, *LPL, ABO, RAI1* and *MC4R*) showed evidence (PPA3 ≥ 0.7) of sharing the same underlying causal variant (Table 4). Overall, we found a total of 44 regions where the PPA3 > 0.7 and both CAD and T2D GWAS studies had some level of genetic association (FDR <1%, Table S8, Figure S6).

**Table 4:** GWAS‐PW results

Chromosome	Start (bp hg19)	End (bp hg19)	PPA Model 3	PPA Model 4	Locus name
6	30,798,168	31,570,931	0.93	0.07	MHC
8	19,492,840	20,060,468	0.99	0.01	*LPL*
9	135,298,917	137,028,444	0.77	0.23	*ABO*
17	16,412,352	18,855,987	0.81	0.18	*RAI1*
18	57,631,234	59,020,370	0.98	0.02	*MC4R*
9	20,464,018	22,205,246	0.00	1.00	9p21^a^

*Note*: Overlapping genome‐wide significant loci in both T2D and CAD with same causal SNP (PPA Model 3 > 0.7). PPA Model 3 is the posterior probability that the genetic associations for both diseases are consistent with a shared causative variant. PPA Model 4 is the posterior probability that the genetic associations for the two diseases are consistent with different causative variants.

Abbreviations: CAD, coronary artery disease; GWAS, genome‐wide association studies; PPA, posterior probability of association; SNP, single‐nucleotide polymorphism; T2D, type 2 diabetes.

^a^ This locus represents an exemplar of a locus containing different T2D and CAD causal variants. Complete set of results in Table S8.

## 4 DISCUSSION

Observational epidemiological studies have consistently reported an increased risk of cardiovascular disease in T2D patients (Einarson et al., 2018; Lautsch et al., 2019). Although some clinical trials indicate a reduction in CAD risk following careful glycaemic control in diabetics (Chiasson et al., 2003; Zinman et al., 2015), others show more complex or contrasting results (Holman et al., 2017). A recent study of insulin‐treated patients suggested how an overall beneficial effect on CAD risk could be partly neutralized due to effects of insulin increasing oxidative stress in arterial vessel walls (Akoumianakis et al., 2020). Some drugs that affect multiple biochemical and cellular pathways, give rise to disparate physiological effects, for instance, empagliflozin is an effective treatment to reduce glucose levels but it is also associated with a lowering of blood pressure to mechanistically entangle the therapeutic benefits in terms of CAD risk (Zinman et al., 2015). Whilst this is clinically welcome, it confounds attempts to understand the direct causal nature of each component of risk reduction, knowledge needed to guide expectations for the impact of new diabetes treatments on cardiovascular disease risk.

To counter this ‘beneficial entanglement’, there are advantages in studying naturally acquired genetic determinants of diabetes and coronary susceptibility as they are allocated randomly during development so are independent of environmental factors that could confound observational associations (Smith & Ebrahim, 2003). However, the explosion in GWAS data has revealed the wide‐spread presence of human pleiotropy (Hackinger & Zeggini, 2017), where a gene influences several seemingly unrelated or loosely related phenotypes; circumstances that need careful consideration here given the complex inter‐relationships of MS traits acting as confounder, mediator or pleiotropic and their clinical sequelae (Sanderson et al., 2019). With that in mind, we use contemporary MR approaches to provide robust causal estimates of the relationship between diabetes and coronary disease risk, with genetic burden estimates ranging from 6% to 9% per log odds unit of T2D susceptibility. Our analyses were all based on T2D genetic instruments that comfortably surpassed by an order of magnitude the ‘rule of thumb’ *F*‐statistic >10 criterion adopted to flag potential weak instrument bias (Burgess & Thompson, 2011).

There have been several previous MR reports to characterize the genetic burden of CAD in type 2 diabetics (Ahmad et al., 2015; Benn et al., 2012; Gan et al., 2019; Jansen et al., 2015; Merino et al., 2017; Ross et al., 2015; Tikkanen et al., 2016; Zhao et al., 2017). For instance, Ross et al. (Ross et al., 2015) reported a substantial causal estimate for T2D (OR = 1.63, 95% CI: 1.23–2.07) on CAD risk in a multivariate model including LDL in addition to the MS traits HDL, TC, TG and BMI with 59 instrumental SNPs that explain 4.6% T2D liability (*F*‐statistic = 87.4 with an effective sample size = 106,953). This represents a predicted relative risk of CAD for a hypothetical 2.72‐fold increase in T2D risk. Applying the same transformation, (Ross et al., 2015) our multivariate model (Table 3) allowing for MS confounding predicts a lower causal estimate with greater precision (OR = 1.42, 95% CI: 1.24–1.62), assuming a population prevalence for T2D and CAD of 10% and 5%, respectively.

Selecting perfect instrumental variables for an idealized MR analysis is perhaps an unrealizable goal given the complex interactions of molecular processes within cells set off by genetic variation. Horizontal pleiotropy (Tyler et al., 2009), originally dubbed mosaic pleiotropy (Hadorn, 1961) implies a shared molecular link between two phenotypes mediated through the presence of a specific allele, an evolutionary efficient and pervasive consequence of molecular multitasking. A simple statistical test for a non‐zero intercept that extends the standard IVW MR method is sensitive to the presence of directional pleiotropy in IVs. This Egger test was significant (*p* < 0.003) for an unfiltered analysis of 224 instruments indicating an imbalance of pleiotropic effects (Bowden et al., 2015; Burgess & Thompson, 2017). Therefore, we undertook weighted median, mode and MR‐PRESSO tests that provide some protection from pleiotropy; these analyses generated consistent estimates of causal effects OR = 1.09 (95% CI: 1.06–1.12). To complement these robust estimates, our modelling of MS‐mediated pleiotropy shrank the MR estimate slightly to OR = 1.07 although only a few are strong (*F*‐statistic > 10) instruments for their respective MS trait.

In the first wave of GWAS in 2007, a novel locus on chromosome 9p21 was reported to show association to both T2D (Diabetes Genetics Initiative of Broad Institute of Harvard and MIT, Lund University, and Novartis Institutes of BioMedical Saxena et al., 2007; Zeggini et al., 2007) and CAD (McPherson et al., 2007). Broadbent et al. (Broadbent et al., 2008) in a fine‐mapping study showed that although the lead associated SNPs were in close physical proximity, they were located on either side of a recombination hotspot and in approximate linkage equilibrium, and regression modelling of comorbid cases confirmed their statistical independence. When we applied *gwas‐pw* to the present GWAS data in a control (i.e., confirmatory) analysis, the 9p21 locus showed a posterior probability of association of 1.0 for Model 4 of independent causal variants (Table 4), confirming the statistical independence of the two genetic signals.

We found that only 5 out of 47 genetically independent loci showing GWS associations to both T2D and CAD had Model 3 posterior probabilities of over 70% for sharing of same causal variant. GWAS has been instrumental in identifying variants associated with diseases and specific biological experiments are needed to pinpoint causal genes. These five loci (MHC, *MC4R, LPL*, *ABO* and *RAI1*) show strong associations with relevant phenotypes and have been well characterized in the literature and, as a result, the likely causal genes are considered known. It has been reported that loss‐of‐function mutations in the coding sequence of *MC4R* gene increases the predisposition to obesity. Conversely, gain‐of‐function variants significantly lower BMI and lower odds of T2D and CAD (Lotta et al., 2019). Protein altering changes to *LPL* gene has been shown to be associated with higher triglyceride levels and presence of CAD (Khera et al., 2017). However, the regulation of this gene is insulin dependent and hence LPL level changes according to insulin levels and sensitivity (Taskinen, 1987). It has been observed in a large prospective cohort that people with O blood group have a lower risk of developing T2D and higher risk with AB+ group (Fagherazzi et al., 2015). Furthermore, the risk of developing CAD is lower in individuals with O blood group (Chen et al., 2016). These GWS loci that are driven by the same causal variant as highlighted in our *gwas‐pw* analysis and reported in the literature suggest a common mediating pathway between the gene and both T2D and CAD. Relaxing the GWS threshold to include FDR <1% loci revealed a significant excess of Model 3 loci, 1.4‐fold more overlaps than expected by chance (Figure S6) providing a valuable resource of potential target regions. Unlike MR approaches where we have the flexibility to assess the relevance of MS traits on the estimated causal effect, *gwas‐pw* only compares regional distribution of summary statistics between two diseases for co‐localization. Functional genomic progress will enable the link between likely causal gene for these loci and how they intersect with different MS traits.

Sullivan et al (Bulik‐Sullivan et al., 2015) reported a cross‐trait LD Score regression correlation between T2D and CAD of 0.39 and with availability of updated summary statistics, we observed a very similar moderate correlation of 0.40. Genetic correlations may be indistinguishable from *signed* pleiotropy (Bulik‐Sullivan et al., 2015), situations where a variant induces a change in two phenotypes in a consistent direction. Our extended MR analysis found that the effects of MS traits on T2D IVs on average, affect CAD risk in a consistent *signed* manner demonstrating the orchestrated effects of T2D variation on MS and CAD.

In summary, our study using large scale GWAS summary statistics based on hundreds of thousands of participants shows that inherited T2D susceptibility is associated with a robust albeit modest increase in CAD susceptibility, indicating a causal link between the two diseases. The link appears to be independent of MS susceptibility with MR estimates that reflect lifelong exposure including a prediabetes phase. Given that the genetic signals observed for common diseases have small to modest effect sizes, even with a significant excess in loci that overlap between the two diseases, the anticipated benefits of diabetes treatments specifically on reducing CAD risk may therefore be similarly modest in scale, with implications for clinical trial design in terms of follow‐up timelines and sample size.

## CONFLICT OF INTERESTS

The authors declare that there are no conflict of interests.

## Supporting information

Supporting information.

Supporting information.

## Data Availability

All data used in this manuscript are publicly available. T2D: https://diagram-consortium.org/downloads.html CAD: https://www.cardiomics.net/download-data Lipids: http://csg.sph.umich.edu/willer/public/lipids2010/ BMI, WHR: https://portals.broadinstitute.org/collaboration/giant/index.php/GIANT_consortium_data_files Fasting glucose: https://magicinvestigators.org/downloads/ Blood pressure: http://ldsc.broadinstitute.org/ldhub/
