# Supplementary material for: Robust estimates of heritable coronary disease risk in individuals with type 2 diabetes
Source: Genet Epidemiol. 2021 Oct 21;46(1):51–62. doi: 10.1002/gepi.22434 (PMC8983061; doi:10.1002/gepi.22434)
Supplement: Supplementary file 1 — Supporting information. [file GEPI-46-51-s002.pdf]

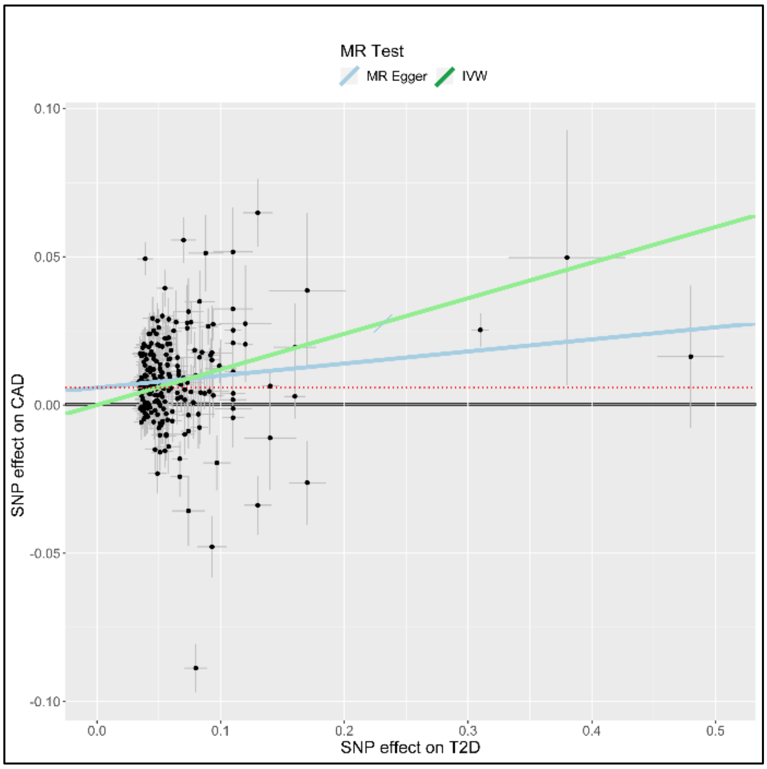

Supplementary Figure 1: Scatter plot of all IVs (n = 224) with T2D effect size vs CAD effect sizes. IVW estimate is the green line, MR egger estimate is the blue line. The egger intercept is the dotted red line.

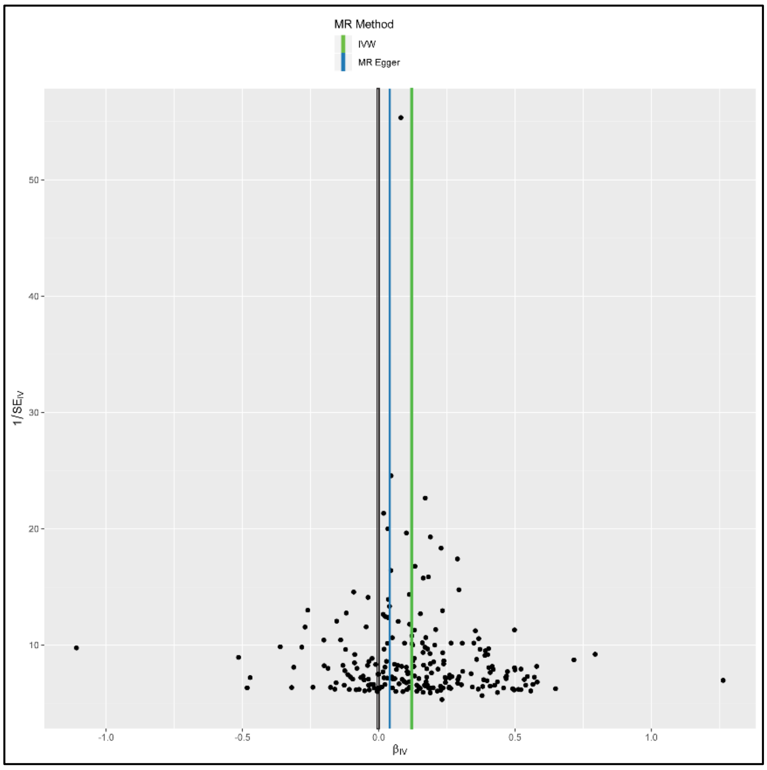

Supplementary Figure 2: Funnel plot of all IVs (n = 224). Instrument strength ($1/SE_{IV}$) against causal estimate on each variant individually (β_{IV}).

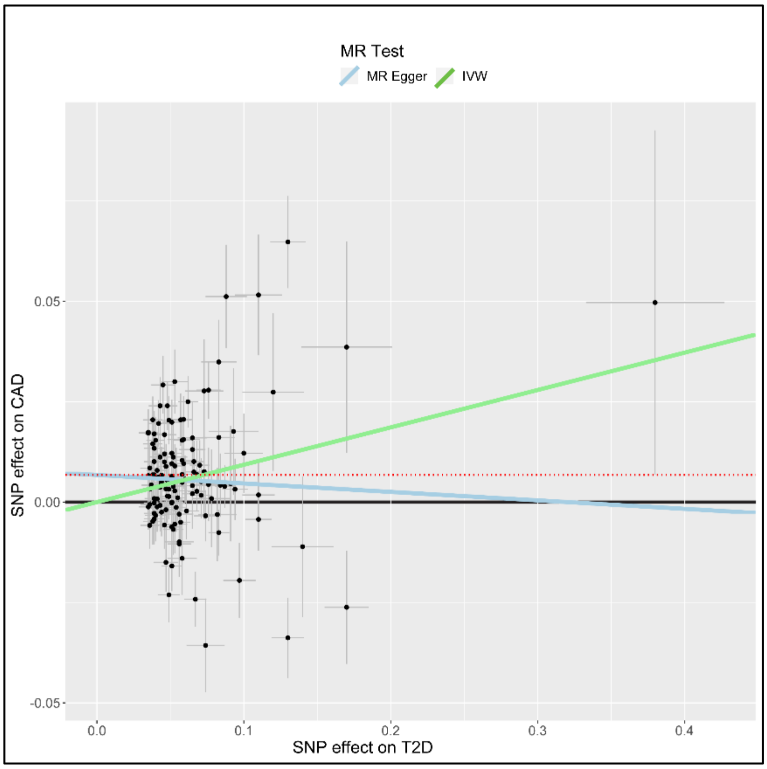

Supplementary figure 3: Scatter plot of IVs with pleiotropic variants removed (n = 131) with T2D effect size vs CAD effect sizes. IVW estimate is the green line, MR egger estimate is the blue line. The egger intercept is the dotted red line.

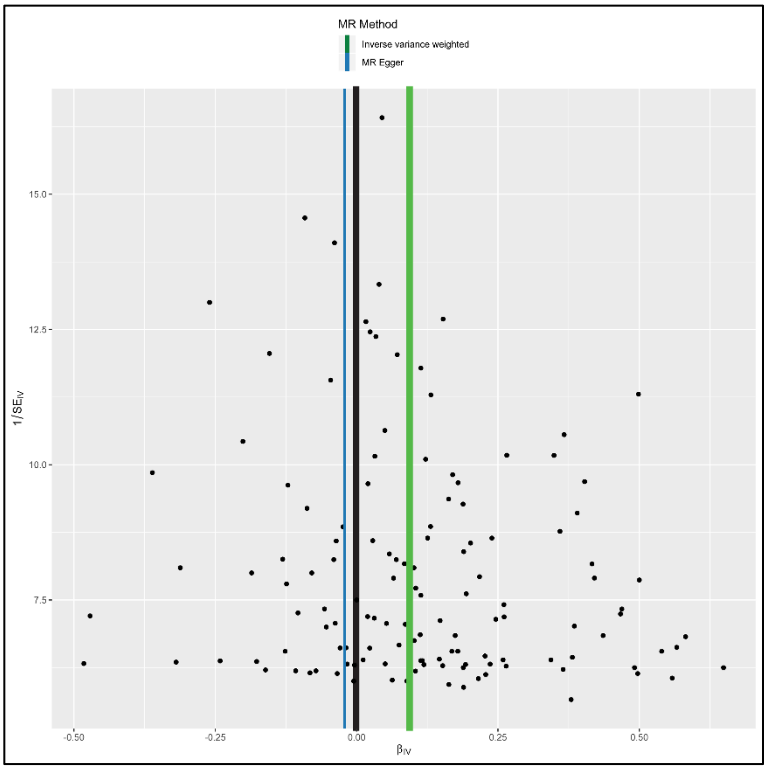

Supplementary figure 4: Funnel plot of IVs with pleiotropic variants removed (n = 131). Instrument strength ($1/SE_{IV}$) against causal estimate on each variant individually (β_{IV}).

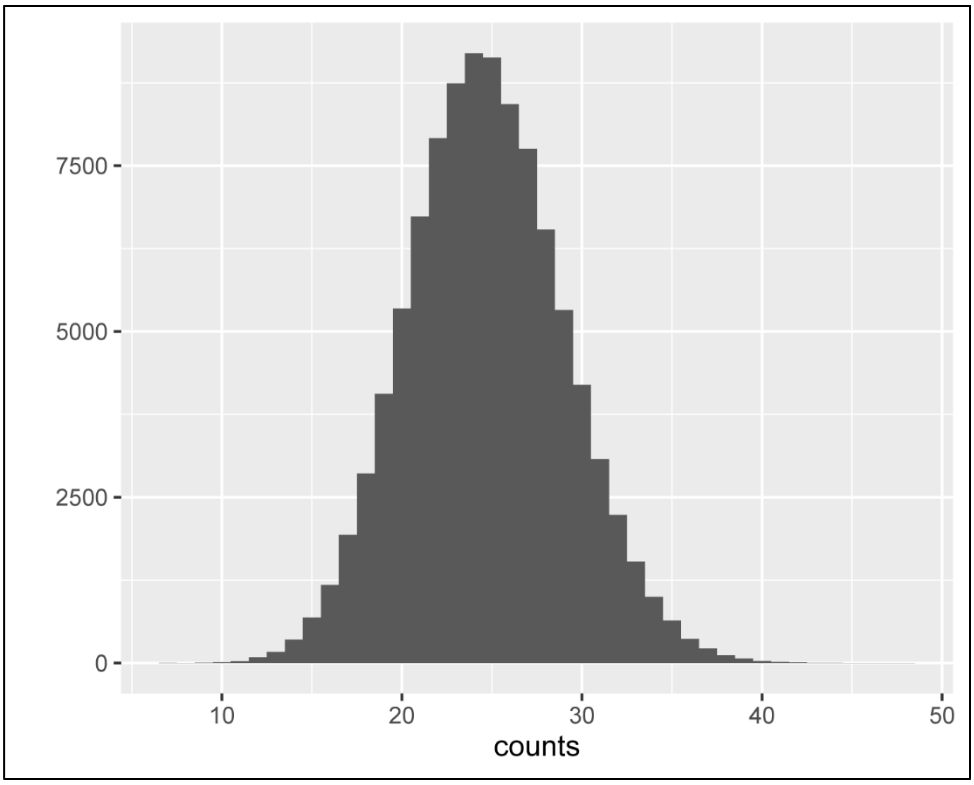

Supplementary figure 5: Histogram of simulated counts of overlaps between T2D and CAD loci. The analysis takes the count of CAD loci (173) and T2D loci (243) and the total number of genomic regions (1703). It randomly assigns the CAD and T2D at locations within the 1703 regions. Then it checks how many of the CAD and T2D loci overlap and returns a count of this. This is repeated 100,000 times.

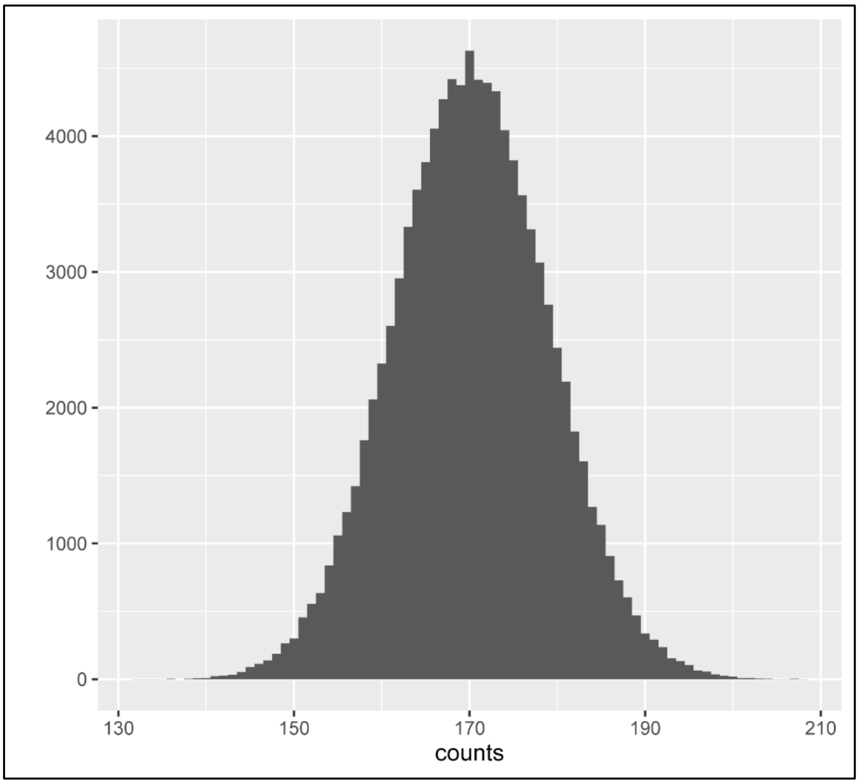

Supplementary figure 6: Histogram of simulated counts of overlaps between T2D and CAD loci at FDR 1%. The analysis takes the count of CAD loci (463) and T2D loci (626) and the total number of genomic regions (1703). It randomly assigns the CAD and T2D at locations within the 1703 regions. Then it checks how many of the CAD and T2D loci overlap and returns a count of this. This is repeated 100,000 times.
